# Effects of Supplementation with Neptune Krill Oil (Euphasia Superba) on Selected Redox Parameters and Pro-Inflammatory Markers in Athletes during Exhaustive Exercise

**DOI:** 10.1515/hukin-2015-0056

**Published:** 2015-10-14

**Authors:** Anna Skarpańska-Stejnborn, Łucja Pilaczyńska-Szcześniak, Piotr Basta, Justyna Foriasz, Jarosław Arlet

## Response

We appreciate Dr. Einar Lied’s comments regarding our article (Skarpańska–Stejnborn et al., 2010). Indeed, we mistakenly used the trade name “Neptune Krill Oil” (Neptune Technologies & Bioresources) instead of “Krill Oil” manufactured by Enzymotec Ltd, India and supplied by Euro-Pharma Alliance Sp. z o.o., Wrocław, as stated in the Material and Methods section. As Dr. E. Lied rightly concluded, misnaming the preparation, especially in the title of the paper, might be misleading for its readers. However, this was not our intention. We mistakenly used the name “Neptune Krill Oil” misinterpreting it as a proper name of the product, rather than as its tradename.

As rightly pointed out by Dr. E. Lied, our study was the first one to analyze the application of krill oil as an attenuator of oxidative stress in athletes. The aim of our study was to verify if the administration of natural supplements with established antioxidant properties, such as krill oil, may attenuate the consequences of oxidative stress induced by exhaustive physical exercise.

Our analysis confirmed that the supplementation with krill oil resulted in a significant reduction of oxidative stress in rowers, as shown by a decrease in the post-exercise damage of erythrocyte lipids in the supplemented group and lack of this beneficial effect in the placebo group. Since both groups of the athletes were exposed to the same training loads and performed the same exercise tests, the difference in the levels of lipid peroxidation products could be interpreted solely as a result of the supplementation.

Another vital issue is the content of active compounds within the supplement given to the athletes. As recommended, we enclose additional data on the contents of various components per 100 g of krill oil supplemented to rowers participating in this study (Table 1).

Another of Dr. E. Lied’s concerns was related to the lack of information on the sources of funding. Our research was supported solely from the statutory funds of our University. Typically, we had not disclosed information on this form of support in our previously published papers, listing the source of funding solely with regard to studies supported from external grants.

We would like to mention that the aforementioned Dr. E. Lied’s comments on our published article have no influence on the results of our research; nevertheless, when considered they may add considerably to the quality of published data.

## Figures and Tables

**Table 1:** Composition of the supplement (Krill Oil) per 100 g of the product

Ingredients	Contents
Phospholipids (g/100g)	42

DHA + EPA (g/100g)	28
DHA	11
EPA	17

Omega 3 (g/100g)	31

Astaxanthin (g/100g)	0.19

Vitamin A (IU/g)	142

